# Supplementary material for: A UMLS-based spell checker for natural language processing in vaccine safety
Source: BMC Med Inform Decis Mak. 2007 Feb 12;7:3. doi: 10.1186/1472-6947-7-3 (PMC1805499; doi:10.1186/1472-6947-7-3)
Supplement: Additional file 13 — RAP application directory. Sets up directory for RAP (RDF application for PHP) source code [file 1472-6947-7-3-S13.gz › rap/doc/phpdoc/__filesource/fsource_dataset__datasetTriXParser.php.html]

xml version="1.0" encoding="iso-8859-1"?


File Source for TriXParser.php


# Source for file TriXParser.php

Documentation is available at TriXParser.php

```
1. <?php
2. // ----------------------------------------------------------------------------------
3. // Class: TriXParser
4. // ----------------------------------------------------------------------------------


8. /**
9. * Temporary implementation of a TriX-Parser
10. * Currently, it doesn't support any namespaces and has problems with typed literals.
11. * So this parser only works with TRIX documents where the default namespace is the TRIX namespace.
12. *
13. * <BR><BR>History:<UL>
14. * <LI>05-07-2005                : First version of this class.</LI>
15. *
16. * @version  V0.9
17. * @author Daniel Westphal (http://www.d-westphal.de)
18. *
19. * @package     dataset
20. * @access    public
21. ***/
22. class TriXParser
23. {
24. /**
25. * Reference to the graphSet
26. *
27. * @var        GraphSet
28. * @access    private
29. */
30. var $graphSet;

32. /**
33. * Constructor
34. * Needs a reference to a graphSet
35. *
36. * @param    GraphSet
37. * @access    public
38. */
39. function TriXParser(&$graphSet)
40. {
41. $this->graphSet=&$graphSet;
42. }

44. /**
45. * Parse an XML string
46. *
47. * @param   string
48. * @access    public
49. */
50. function parseString($string)
51. {
52. $this->_populateGraphSet(simplexml_load_string($string));
53. }

55. /**
56. * Parse from a file
57. *
58. * @param   string
59. * @access    public
60. */
61. function parseFile($pathToFile)
62. {
63. $this->_populateGraphSet(simplexml_load_file($pathToFile));
64. }

66. /**
67. * Populates the graphSet with namedGraphs and triples.
68. *
69. * @param   object simpleXMLModel  $xmlModel
70. * @access    private
71. */
72. function _populateGraphSet(&$xmlModel)
73. {
74. foreach ($xmlModel->graph as $graph)
75. {
76. if (isset($graph->uri))
77. {
78. $graphName=(string)$graph->uri;
79. } else
80. {
81. $graphName="urn:" . (string)uniqid();
82. }
83. $namedGraph=&$this->graphSet->createGraph($graphName);

85. foreach ($graph->triple as $triple)
86. {
87. $tripleCount=0;
88. $tripleArray=array();
89. foreach ($triple->children() as $tag => $value)
90. {
91. $tripleArray[$tripleCount++]=$this->_element2Resource((string)$tag,$value);
92. };
93. $namedGraph->add(new Statement($tripleArray[0],$tripleArray[1],$tripleArray[2]));
94. };
95. };
96. }

98. /**
99. * return a mathing resource tyoe
100. *
101. * @param   string
102. * @param   object simpleXMLNode $value
103. * @access    private
104. */
105. function _element2Resource($tag,$value)
106. {
107. switch ($tag)
108. {
109. case 'uri':
110. return new Resource((string)$value);
111. break;

113. case 'id':
114. return new BlankNode((string)$value);
115. break;

117. case 'typedLiteral':
118. $literal=new Literal((string)$value);
119. $literal->setDatatype((string)$value['datatype']);
120. return $literal;
121. break;

123. case 'plainLiteral':
124. $literal=new Literal((string)$value);
125. if(isset($value['xml:lang']))
126. $literal->setLanguage((string)$value['xml:lang']);
127. return $literal;
128. break;
129. }
130. }
131. }
132. ?>
```

Documentation generated on Mon, 4 Jul 2005 17:14:53 +0200 by phpDocumentor 1.3.0RC3
